# A predictive score for atrial fibrillation in poststroke patients

**DOI:** 10.1055/s-0044-1788271

**Published:** 2024-08-15

**Authors:** Caroliny Trevisan Teixeira, Vanessa Rizelio, Alexandre Robles, Levi Coelho Maia Barros, Gisele Sampaio Silva, João Brainer Clares de Andrade

**Affiliations:** 1 Hospital Instituto de Neurologia de Curitiba, Curitiba PR, Brazil.; 2 Centro Universitário São Camilo, São Paulo SP, Brazil.; 3 Universidade Estadual do Ceará, Fortaleza CE, Brazil.; 4 Universidade Federal de São Paulo, São Paulo SP, Brazil.; 5 Instituto Tecnológico de Aeronáutica, São José dos Campos SP, Brazil.; 6 Hospital Israelita Albert Einstein, Organização de Pesquisa Acadêmica, São Paulo SP, Brazil.

**Keywords:** Ischemic Stroke, Atrial Fibrillation, Prognosis, AVC Isquêmico, Fibrilação Atrial, Prognóstico

## Abstract

**Background** Atrial fibrillation (AF) is a risk factor for cerebral ischemia. Identifying the presence of AF, especially in paroxysmal cases, may take time and lacks clear support in the literature regarding the optimal investigative approach; in resource-limited settings, identifying a higher-risk group for AF can assist in planning further investigation.

**Objective** To develop a scoring tool to predict the risk of incident AF in the poststroke follow-up.

**Methods** A retrospective longitudinal study with data collected from electronic medical records of patients hospitalized and followed up for cerebral ischemia from 2014 to 2021 at a tertiary stroke center. Demographic, clinical, laboratory, electrocardiogram, and echocardiogram data, as well as neuroimaging data, were collected. Stepwise logistic regression was employed to identify associated variables. A score with integer numbers was created based on beta coefficients. Calibration and validation were performed to evaluate accuracy.

**Results** We included 872 patients in the final analysis. The score was created with left atrial diameter ≥ 42 mm (2 points), age ≥ 70 years (1 point), presence of septal aneurysm (2 points), and score ≥ 6 points at admission on the National Institutes of Health Stroke Scale (NIHSS; 1 point). The score ranges from 0 to 6. Patients with a score ≥ 2 points had a fivefold increased risk of having AF detected in the follow-up. The area under the curve (AUC) was of 0.77 (0.72–0.85).

**Conclusion** We were able structure an accurate risk score tool for incident AF, which could be validated in multicenter samples in future studies.

## Resumo

**Antecedentes** Fibrilação atrial (FA) é um fator de risco para isquemia cerebral. Identificar a presença de FA, especialmente em casos paroxísticos, pode demandar tempo, e não há fundamentos claros na literatura quanto ao melhor método de proceder à investigação; em locais de parcos recursos, identificar um grupo de mais alto risco de FA pode auxiliar no planejamento da investigação complementar.

**Objetivo** Desenvolver uma ferramenta de escore para prever o risco de FA no acompanhamento após acidente vascular cerebral (AVC).

**Métodos** Estudo longitudinal retrospectivo, com dados coletados dos prontuários eletrônicos de pacientes hospitalizados e acompanhados ambulatorialmente por isquemia cerebral, de 2014 a 2021, em um centro de AVC terciário. Foram coleados dados demográficos, clínicos, laboratoriais, de eletrocardiograma e ecocardiograma, além de dados de neuroimagem. Mediante uma regressão logística por *stepwise* , foram identificadas variáveis associadas. Um escore com números inteiros foi criado com base nos coeficientes beta. Calibração e validação foram realizadas para avaliar a precisão.

**Resultados** Foram incluídos 872 pacientes na análise final. O escore foi criado com diâmetro de átrio esquerdo ≥ 42 mm (2 pontos), idade ≥ 70 anos (1 ponto), presença de aneurisma septal (2 pontos) e pontuação à admissão ≥ 6 na escala de AVC dos National Institutes of Health (National Institutes of Health Stroke Scale, NIHSS, em inglês; 1 ponto). O escore tem pontuação que varia de 0 a 6. Pacientes com escore ≥ 2 pontos tiveram cinco vezes mais risco de terem FA detectada no acompanhamento. A área sob a curva ( *area under curve* , AUC, em inglês) foi de 0.77 (0.72–0.85).

**Conclusão** Pudemos estruturar uma ferramenta precisa de escore de risco de FA, a qual poderá ser validada em amostras multicêntricas em estudos futuros.

## INTRODUCTION

Strokes are responsible for approximately 10% of all deaths worldwide, being one of the major preventable causes of morbimortality nowadays. 1 One of the chief strategies for stroke prevention is assessment of heart rhythm. In this regard, atrial fibrillation (AF) is the most identified etiology of ischemic stroke. Additionally, about 30% of patients initially classified with cryptogenic stroke are later diagnosed with AF. 2

The term *atrial heart disease* has been used to identify patients with left atrium structural and/or functional abnormalities, potentially leading to paroxysmal AF. 3 4 5 Recent evidence 6 7 8 suggests that atrial pathologies could be responsible for thromboembolic events even before AF development.

Regrettably, heart rhythm assessment could be troublesome, often relying on devices not readily available, especially in limited-resource settings. The extensive duration in some cardiac rhythm monitoring strategies also poses a challenge for secondary stroke prevention. 3 4 5 6 7 8 9 10

Considering this context, a better selection of stroke patients eligible for longer cardiac rhythm monitoring based on their risk for thromboembolism and AF could address the drawbacks related to this diagnostic tool. 11 Including an assessment of the atrial thromboembolic substrate could also increase the detection of patients at a high risk of having recurrent stroke. 11

Therefore, we aimed to develop an incident AF predictive risk score based on available clinical, laboratorial, imaging, and cardiac findings after acute ischemic stroke. The proposed score could later help providers to reduce the stroke burden through tailored strategies in high-risk patients.

## METHODS

### Design

In the present retrospective cohort study, data was collected from patients diagnosed with acute ischemic stroke and followed up at a tertiary stroke center in the city of Curitiba, Brazil, from January 2014 to July 2021.

### Data collection

Demographic, clinical, laboratorial, neuroimaging, and cardiac monitoring data were searched through the service's database and extracted from electronic medical records. Patients with previous AF diagnosis, lacunar syndrome presentation on admission or insufficient data were excluded from the analysis.

To assess atrial stress, we selected evaluated left atrium size, left ventricular ejection fraction (LVEF), presence of interatrial septum aneurysm, and presence of left atrium thrombus identified on echocardiography as surrogates. Atrial and ventricular arrhythmias were assessed through 24-hour Holter monitoring according to the neurologist's decision.

### Outcomes

The primary endpoint was the time until the development of incident AF, defined as the first occurrence of AF based on a hospital diagnosis, physician medical records, or related hospital procedures using the coding of the International Classification of Diseases, 10th edition (ICD-10). Atrial fibrillation was defined based on an electrocardiogram (ECG) or Holter recording. The inclusion criteria for an AF case were limited to inpatients with AF diagnosis confirmed on admission and discharge. The accuracy of the diagnosis was previously validated using sensitivity analysis. Hospital inpatient and procedure data were identified through linkage with local electronic medical records. All patients included in the final analysis underwent a minimum follow-up of 6 months poststroke and at least one 24-hour cardiac monitoring session during the period. Patients not meeting these criteria were excluded from the final analysis.

We defined follow-up duration as the time from the baseline exam to incident AF, death, or the last available follow-up contact, whichever occurred first.

### Statistical analysis

Data were expressed as mean and standard deviation (SD) values for the continuous variables, and as proportions for the categorical variables. An unpaired, two-tailed Student *t* -test was used to analyze the continuous variables, and differences among the nominal variables were compared using the Chi-squared test.

The variables were independently evaluated in terms of their relationship with AF occurrence during the follow-up, and they were removed stepwise from the model when the *p* -value exceeded 0.10. The variables with a *p* -value lower than 0.05 in the final model were considered significant contributors to prediction, and we have reported the net odds ratio (OR), 95% confidence interval (95%CI), and *p* -value for these variables. The variables in the final model were tested for interactions, if any.

The calibration of the model was assessed with the Hosmer-Lemeshow test. The independent predictive factors identified in the logistic regression analyses were then used to develop a predictive grading score for AF during follow-up. The score of each variable was defined based on coefficients of the multivariable logistic equation by rounding to the nearest integer. Bootstrapping was used to reduce bias in the performance estimates. We assessed the discrimination of the score using the area under the receiver operating characteristic (AUC-ROC) curve. The optimal cutoff point of our score was defined using the Youden Index. From the final model, the variables had an integer number attributed based on their beta coefficients, as suggested by the literature. 12 13 Statistical significance was set at *p* < 0.05, and all statistical analyses were conducted using the SPSS Statistics for Windows (SPSS Inc., Chicago, IL, United States) software, version 17.0.

### Standard protocol approvals, registrations, patient consent, and data availability

The present study received approval from the Institutional Review Board (IRB) at the Hospital Instituto de Neurologia de Curitiba (Brazil), ensuring compliance with all Brazilian ethical guidelines for research. The IRB granted an exemption from obtaining informed consent for the validation group, considering the retrospective nature of data collection. The research findings, including all relevant data, are systematically presented in the study's tables and figures. Moreover, any data that have been anonymized are available for public access, facilitating transparency and further research in the field.

## RESULTS

Of the 1,025 confirmed cases of ischemic stroke admitted to the service from 2014 to 2021, 872 patients were included in the final analysis; 70 of them were excluded due to previous diagnosis of AF, and 83 were also excluded due to insufficient data on the medical records.

Incident AF was observed in 79 (9.05%) patients after discharge from the follow-up, at a median time of 12 months, and 5 (0.6%) performed 24h-cardiac monitoring.

The demographic, clinical, neuroimaging, and cardiac assessment variables are described in Table 1 . The median age was of 72 (range: 61–79) years, and 481 (55%) patients were men. Regarding comorbid conditions, 555 (64%) patients had hypertension, 261 (30%) had diabetes, 173 (20%) had already had a stroke, 160 (18%) patients were smokers, and 95 (11%) had already had acute myocardial infarction (AMI). As for the etiological stroke classification of the Trial of Org 10172 in Acute Stroke Treatment (TOAST), considering all included patients, 6.5% were classified as cardioembolic, 25%, atheroembolic/large vessels, 7.3%, secondary to other causes, 21%, small vessels, and 41%, cryptogenic. Of the patients classified as cryptogenic, 202 (55%) met criteria 7 14 for embolic stroke of undetermined source (ESUS).

**Table 1:** Baseline characteristics of the study sample

Variables	Patients (N = 872)	AF detected during follow-up (n = 170)	AF not detected during follow-up (n = 702)	*p* -value
**Age in years: median (IQR)**	72 (61–79)	78 (68–84)	70 (59–78)	**< 0.001**
**Male patients: %**	50	53.5	49.6	0.56
**Weight in kilos: mean ± SD**	74.7(±15.2)	74.5(±14.2)	74.9(±15.4)	0.85
**NIHSS score upon admission (points): median (IQR)**	2 (1–4)	4 (2–7)	2 (0–4)	**< 0.001**
**Neuroimaging**				
Bilateral involvement: %	11.7	11.9	11.7	0.96
Cortical lesion: %	62.4	11.7	7.2	**0.03**
Grade on the Fasekas scale: %				**0.008**
0	8	8.1	7.1	
1	34.7	36.4	17.8	
2	28.6	28.6	28.5	
3	28.6	26.5	46.4	
PC-ASPECTS (points): median (IQR)	9 (8–10)	9 (9–10)	9 (8–9)	0.21
ASPECTS (points): median (IQR)	10 (9–10)	9 (9–10)	9 (9–10)	0.71
**Laboratory**				
Blood glucose at admission (mg/dL): median (IQR)	113 (96–142)	118 (101–150)	114 (97–143)	0.30
Platelet count (cells/mm ^3^ ): median (IQR)	201,000 (169,200–244,600)	187,300 (143,800–243,200)	201,700 (170,900–246,400)	**0.03**
LDL (mg/dL): mean ± SD	104.1(±39.6)	94.5(±39.9)	106.9(±40.4)	**0.01**
GFR (mL/min/1.73m ^2^ ): mean ± SD	73(±33.7)	64(±30.5)	75(±34)	**0.03**
**Comorbidities**				
Arterial hypertension: %	70.7	70.2	70.9	0.37
Diabetes mellitus: %	32.3	33.3	32.2	0.78
Dyslipidemia: %	46.3	57.1	45	0.08
Coronaropathy: %	12.2	16.6	11.7	0.36
Previous stroke: %	22.4	20.3	22.8	0.67
Prior TIA: %	3	4.8	2.9	0.3
**Cardiac measurements**				
LA size (mm): median (IQR)	38 (34–40)	43 (38–47)	37 (33–40)	**< 0.001**
LVEF (%): median (IQR)	67 (63–70)	65.5 (58–69)	67 (64–70)	**0.004**
High number of extrasystoles: %	19.5	21.4	19.2	**0.02**
Atrial tachycardia: %	2.8	2.8	0	0.15
PFO: %	13.4	6	14.3	**0.04**
Presence of atrial septal aneurysm: %	3.4	8.4	2.8	**0.01**
**In-hospital treatment, classifications, and outcome**				
IV thrombolysis: %	10.3	17.8	9.4	0.06
Mechanical thrombectomy: %	3.1	2.4	3.2	0.81
SSS-TOAST undetermined classification: %	26.3	26.5	24.1	0.63
Modified Rankin Scale at discharge (points): median (IQR)	1 (0–2)	1 (0–2)	1 (1–3)	**0.008**

Abbreviations: ASPECTS, Alberta stroke programme early CT score; GFR, glomerular filtration rate; IQR, interquartile range; IV, intravenous; LA, left atrium; LDL, low-density lipoprotein; LVEF, left ventricular ejection fraction; NIHSS, National Institutes of Health Stroke Scale; PC-ASPECTS, Posterior Circulation – Acute Stroke Prognosis Early Computed Tomography Scores; PFO, patent foramen ovale; SD, standard deviation; SSS-TOAST, Stop Stroke Study – Trial of ORG 10172 in Acute Stroke Treatment; TIA, transient ischemic attack.

Atrial disease markers were present in 170/872 patients (20%): 146 patients had left atrium enlargement, 20 patients had septal aneurysm, and 4 patients had a thrombus in the left atrial appendage.

From the final stepwise logistic regression model, left atrial size ≥ 42 mm, age ≥ 70 years old, presence of interatrial septal aneurysm, and score on the National Institutes of Health Stroke Scale (NIHSS) ≥ 6 points upon admission were associated with the risk of developing AF, as demonstrated in Table 2 .

**Table 2:** Adjusted logistic regression model from the stepwise method

Variable	Beta-coefficient	OR	95%CI	*p* -value
Age ≥ 70 years	0.95	2.60	1.46–4.63	0.001
NIHSS score upon admission ≥ 6 points	0.74	2.10	1.20–3.67	0.009
Left atrium size ≥ 42 mm	1.58	4.87	2.89–8.20	< 0.001
Presence of interatrial septal aneurysm	1.56	4.77	1.71–13.25	0.003

Abbreviations: 95%CI, 95% confidence interval; NIHSS, National Institutes of Health Stroke Scale; OR, odds ratio.

Notes: Cox and Snell: 0.097; Nagelkerke R ^2^ : 0.193; Hosmer-Lemeshow Chi-squared test: 4.29 ( *p* = 0.36).

**Figure 1:**
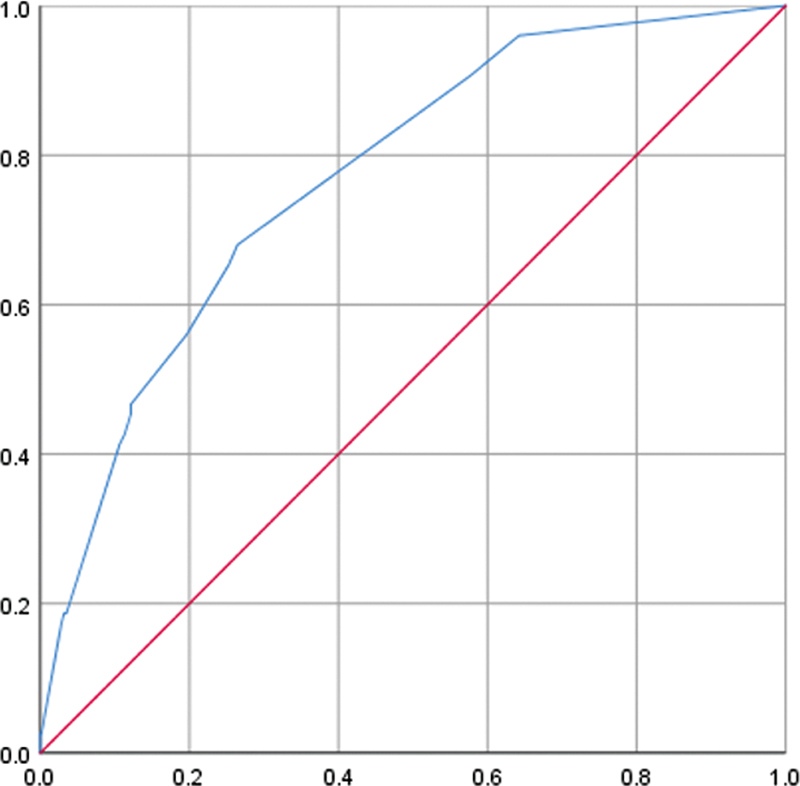
Notes: Y axis – specificity; X axis – sensitivity. Analysis of the area under the receiver operating characteristic curve (AUC-ROC).

When grouped together in a ROC curve, these variables displayed an AUC of 0.77 (0.72–0.82; SD: ± 0.027; *p* < 0.001), as displayed in Figure 1 .

A corresponding score was assigned to each related variable, obtained through their beta coefficients in the final adjusted model, as shown in Table 3 . The cut-off point for AF identification was then established as ≥ 2.

**Table 3:** Predictive score for atrial fibrillation during follow-up

Variable	Point
Age ≥ 70 years	1
NIHss score upon admission ≥ 6 points	1
Left atrium size ≥ 42 mm	2
Presence of septal aneurysm	2
Total	0-6

Abbreviation: NIHSS, National Institutes of Health Stroke Scale.

**Figure 2:**
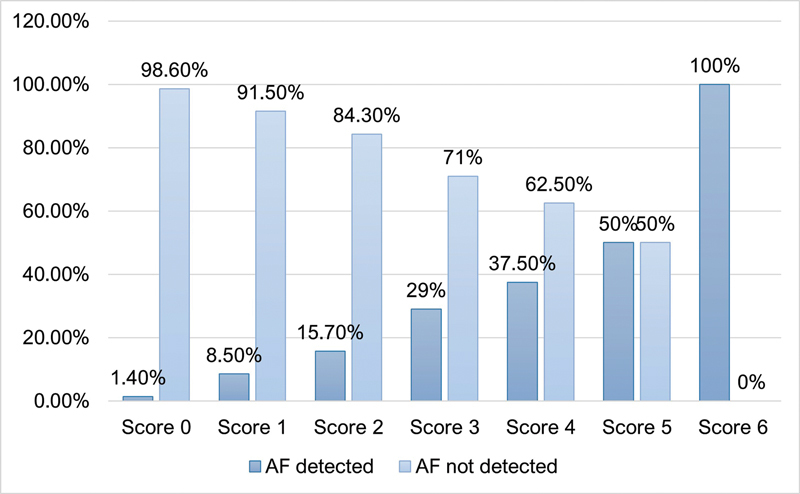
Distribution of cases of atrial fibrillation (AF) during follow-up according to score.

Figure 2 displays the proportion of patients with newly-diagnosed AF in the follow-up period compared to patients without AF, according to the score obtained. A risk score of 6 corresponded to 100% of patients with AF during the follow-up. Based on the adjusted analysis, each point added to the score increased the risk of having AF during the follow-up by 2.3 times (OR: 2.3; 95%CI: 1.9–2.8; *p* -value < 0.001). Moreover, there was a 6.7-fold increased risk of AF occurrence if the score was ≥ 2 (OR: 6.7; 95%CI: 3.9–11.2; *p* < 0.001).

Due to the retrospective design of the study, we observed that there was no consistent pattern in the timing of Holter monitor requests, which ranged from monthly exams to patients who underwent the exam every six months – according to the personal decision of our local staff. For patients with markers of atrial myopathies and a high-risk score, our local protocol recommends conducting the exam more frequently to facilitate the diagnosis of AF or atrial flutter.

Our proposed score is freely available at http://www.afibrisk.net .

## DISCUSSION

According to our data, demographic, clinical, and atrial disease markers may be accurately associated with the occurrence of newly-diagnosed AF during the follow-up for stroke. Left atrium size ≥ 42 mm, age ≥ 70 years old, presence of atrium septal aneurysm, and NIHSS score ≥ 6 points on admission demonstrated a statistically significant correlation with our primary endpoint. Atrial disease markers were present in 170 patients, comprising 20% of our total population.

Biomarkers of atrial dysfunction have been associated with increased risk of ischemic stroke. 14 Almost 65% of the patients with cryptogenic stroke have atrial heart disease, demonstrated by the presence of at least one of the biomarkers. 7 In most cases, left atrial disease is previously unknown to the patient and/or treating physician, and patients with markers of atrial heart disease may experience embolic events even in the absence of AF. 7

According to our database, the diagnosis of AF was established after an average of 8 months of follow-up, which is consistent with the current literature. 14 15 16 In the CRYSTAL AF study, 11 for example, the analysis of the time until the detection of AF was of approximately 6 to 12 months, and systematic reviews evaluating the identification of AF with external ECG monitoring in patients after cryptogenic stroke showed an estimated time ranging from 5 to 12 months. 17 18

Among the study variables associated with AF detection during follow-up, age ≥ 70 years is a widely known risk factor, with AF prevalence reaching 6% of the population over 65 years old. 19 20 21 Increased stroke severity, assessed by the NIHSS, also presented a strong correlation to AF development, possibly because cardioembolic events are often associated with greater vascular occlusions and greater brain area involved. 19 20 22 An interesting finding was that the presence of atrial septal aneurysm was associated with increased risk of AF during follow-up. Atrial septal aneurysm may be an arrhythmogenic focus and may be associated with a higher incidence of atrial arrhythmias, including AF. Nonetheless, few studies on this topic are available. 22 23 24 25

Multiple strategies were previously designed to predict new AF diagnoses in distinct clinical settings and patient populations, some of them incorporating machine-learning processes. 17 20 Although some were exclusively based on ECG findings, 20 21 most of them incorporated a myriad of demographic, clinical, lifestyle, cardiac, and laboratorial markers to create a single predicting tool for improved accuracy. 26 27 28 29 30 31 Notwithstanding, none of these methods were specifically validated on the ischemic stroke population. Our score innovates by incorporating clinical, neuroimaging and echocardiographic findings to create a score that is simple to use and applicable at bedside.

The use of an easy-to-apply score renders AF risk factor assessment more approachable, favoring patient stratification and influencing follow-up management. It is also worth mentioning that, in the event of an acute ischemic stroke, the presence of atrial disease markers, even if AF is absent in the acute phase, should lead to a more thorough and personalized investigation of the cardiac rhythm, with greater frequency of consultations and Holter monitoring requests.

Our proposed score has an accuracy similar to that reported in the literature, being superior to the well-established CHADS2, CHADS2-VASc, 30 C2HEST, 29 ASA, 11 ARIC, 32 and MHS scores. 33 It has the same accuracy as the score derived from the Framingham study, 26 but lower accuracy compared to the HARMS2-AF 24 and STAF 34 scores.

Among the limitations to the present study, a significant drawback is the non-standardization of the time for Holter request, which was left to the discretion of the patient's follow-up physician. Another limitation was the absence of N-terminal pro-brain natriuretic peptide (NT-proBNP) as a laboratory marker for AF in the present analysis, as it was not routinely performed in our center. Additionally, the current study focused on developing a follow-up AF risk score tool, rather than directly assessing whether early detection could be associated with improved stroke outcomes. The retrospective nature of the study is also considered a limiting factor, because it relied on data from electronic medical records throughout the years, making it prone to multiple biases. Finally, we did not validate our score in an external population. Developed at a single center, our score is based on homogeneous clinical and demographic baseline aspects – and this characteristic may limit the generalization of our results. Further multicentric prospective studies could later improve the accuracy of our risk score.

In conclusion, a predictive score consisting of a left atrium size ≥ 42 mm, age ≥ 70 years, NIHSS ≥ 6 points, and the presence of an interatrial septal aneurysm, accurately predicted the occurrence of AF during the follow-up of stroke patients. Our results also suggest the development of a tool that could be subsequently validated in larger samples, potentially influencing the etiological investigation and follow-up management by identifying high-risk AF patients based on in-hospital data.
